# Novel *STAT1* Alleles in Otherwise Healthy Patients with Mycobacterial Disease

**DOI:** 10.1371/journal.pgen.0020131

**Published:** 2006-08-18

**Authors:** Ariane Chapgier, Stéphanie Boisson-Dupuis, Emmanuelle Jouanguy, Guillaume Vogt, Jacqueline Feinberg, Ada Prochnicka-Chalufour, Armanda Casrouge, Kun Yang, Claire Soudais, Claire Fieschi, Orchidée Filipe Santos, Jacinta Bustamante, Capucine Picard, Ludovic de Beaucoudrey, Jean-François Emile, Peter D Arkwright, Robert D Schreiber, Claudia Rolinck-Werninghaus, Angela Rösen-Wolff, Klaus Magdorf, Joachim Roesler, Jean-Laurent Casanova

**Affiliations:** 1 Laboratory of Human Genetics of Infectious Diseases, University of Paris René Descartes, INSERM U550, Necker Medical School, Paris, France, European Union; 2 French-Chinese Laboratory of Genetics, Ruijin Hospital, Shanghai II University, Shanghai, People's Republic of China; 3 Laboratory of MNR of Biomolecules, CNRS URA2185, Pasteur Institute, Paris, France, European Union; 4 Service of Clinical Immunology, Saint Louis Hospital, Paris, France, European Union; 5 Center for the Study of Immunodeficiences, Necker Hospital, Paris, France, European Union; 6 Department of Pathology, Ambroise Paré Hospital, Boulogne, France, European Union; 7 University of Manchester, Manchester, United Kingdom; 8 Department of Pathology and Immunology, Washington University, Saint Louis, Missouri, United States of America; 9 Department of Pediatric Pneumology and Immunology, Charité, Humboldt University of Berlin, Berlin, Germany; 10 Department of Pediatrics, University Clinic Carl Gustav Carus, Dresden, Germany; 11 Pediatric Immunology Hematology Unit, Necker Hospital, Paris, France, European Union; MRC Human Genetics Unit, United Kingdom

## Abstract

The transcription factor signal transducer and activator of transcription-1 (STAT1) plays a key role in immunity against mycobacterial and viral infections. Here, we characterize three human *STAT1* germline alleles from otherwise healthy patients with mycobacterial disease. The previously reported L706S, like the novel Q463H and E320Q alleles, are intrinsically deleterious for both interferon gamma (IFNG)–induced gamma-activating factor–mediated immunity and interferon alpha (IFNA)–induced interferon-stimulated genes factor 3–mediated immunity, as shown in STAT1-deficient cells transfected with the corresponding alleles. Their phenotypic effects are however mediated by different molecular mechanisms, L706S affecting STAT1 phosphorylation and Q463H and E320Q affecting STAT1 DNA-binding activity. Heterozygous patients display specifically impaired IFNG-induced gamma-activating factor–mediated immunity, resulting in susceptibility to mycobacteria. Indeed, IFNA-induced interferon-stimulated genes factor 3–mediated immunity is not affected, and these patients are not particularly susceptible to viral disease, unlike patients homozygous for other, equally deleterious *STAT1* mutations recessive for both phenotypes. The three *STAT1* alleles are therefore dominant for IFNG-mediated antimycobacterial immunity but recessive for IFNA-mediated antiviral immunity at the cellular and clinical levels. These *STAT1* alleles define two forms of dominant STAT1 deficiency, depending on whether the mutations impair STAT1 phosphorylation or DNA binding.

## Synopsis

Mendelian susceptibility to mycobacterial disease is a rare syndrome. It is defined by the occurrence of severe disease caused by low virulence mycobacteria in otherwise healthy individuals, in whom antiviral immune response is not affected. Eleven known genetic defects, affecting five genes, have been involved in this type of deficient response to infection, involving immune-mediator molecules IL12 and interferon gamma: *IL12B, IL12RB1, IFNGR1, IFNGR2,* and *STAT1.* The signal transducer and activator of transcription-1 (STAT1) amino acid change L706S was previously shown to cause disease by impairing STAT1 phosphorylation. Here, we report two new *STAT1* mutations that impair STAT1 DNA-binding activity. We show, by functional analysis of the three *STAT1* mutant alleles, that they are intrinsically deleterious for both interferon gamma–induced antimycobacterial immunity, which is mediated through gamma-activated factor and for interferon alpha–induced antiviral immunity, which is mediated through interferon-stimulated genes factor 3. Interestingly, the three alleles are dominant for interferon gamma–induced gamma-activated factor–mediated antimycobacterial immunity, but recessive for interferon alpha–induced interferon-stimulated genes factor 3–mediated antiviral immunity at the cellular and clinical levels. These two new *STAT1* alleles, which affect the binding of STAT1 to DNA, define distinct novel genetic causes of Mendelian susceptibility to mycobacterial disease and provide further insight into the molecular mechanism of disease.

## Introduction

Mendelian susceptibility to mycobacterial disease (MSMD) is characterized by the occurrence of clinical disease caused by weakly virulent mycobacteria in otherwise healthy individuals (reviewed in [1,2]). This syndrome covers a broad range of clinical phenotypes, reflecting the diversity of environmental and host factors involved, notably the underlying genetic lesions. The five genes known to cause this syndrome are involved in IL12/23-dependent interferon gamma (IFNG)–mediated immunity. Two genes control the production of IFNG: *IL12B,* encoding the p40 subunit of IL12 and IL23, and *IL12RB1,* encoding the β_1_ chain of the IL12 and IL23 receptors (IL12RB1). Three genes control the response to IFNG: *IFNGR1* and *IFNGR2,* encoding the IFNG receptor (IFNGR) chains, and *STAT1,* encoding the signal transducer and activator of transcription-1 (STAT1). Allelic heterogeneity results in a total of 11 inherited disorders (Table 1): recessive complete IL12p40 [3,4] and IL12RB1 deficiency with [5] or without [6–8] surface-expressed receptors, recessive complete IFNGR1 deficiency with [9] or without [10,11] surface-expressed receptors, dominant [12] or recessive [13] partial IFNGR1 deficiency, recessive complete IFNGR2 deficiency with [14] or without [15] surface-expressed receptors, recessive partial IFNGR2 deficiency [16], and dominant partial STAT1 deficiency [17]. Complete IFNGR1 and IFNGR2 deficiencies run a more severe clinical course than the other defects, which are associated with residual IFNG-mediated immunity [1,2,18,19].

**Table 1:**
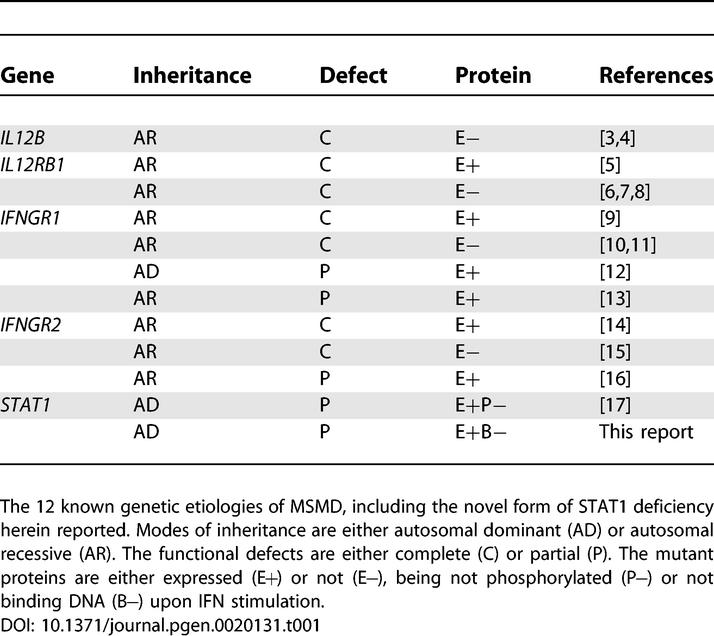
Genetic Etiology of MSMD

**Figure 1:**
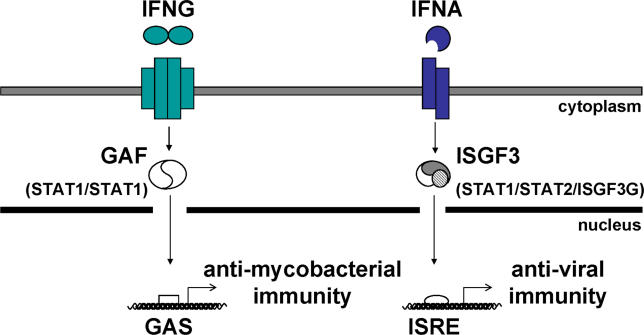
Pathways of IFNG-Induced GAF-Mediated Immunity and IFNA-Induced ISGF3-Mediated Immunity Patients homozygous for null STAT1 mutations [36,37] suffer from both mycobacterial and viral diseases. Patients heterozygous for STAT1 mutations L706S, Q463H, and E320Q suffer from mycobacterial but not viral diseases ([17] and this report).

The binding of homodimeric IFNG to its tetrameric receptor leads to the activation of constitutively associated Janus kinases 1 and 2 (JAK1 and JAK2), which then phosphorylate tyrosine residues in the intracellular part of IFNGR1 (reviewed in [20–25]; Figure 1). Most latent STAT1 molecules reside in the cytosol as preassociated unphosphorylated homodimers [26–29]. Upon IFNG stimulation, unphosphorylated STAT1 molecules are directly recruited to IFNGR1 docking sites (centered on phosphorylated Y440) [26,30]. They are then phosphorylated at Y701 and released into the cytosol as phosphorylated STAT1 homodimers, forming gamma-activating factors (GAFs), which are translocated to the nucleus [31]. GAF binds gamma-activating sequences (GASs) present in the promoters of target genes [32]. Following monomeric interferon alpha (IFNA) stimulation, STAT2 is recruited to the phosphorylated IFNAR1 chain of the heterodimeric IFNAR and is itself also phosphorylated by JAK1 and TYK2 (reviewed in [20,22–25,33]; Figure 1). This leads to the phosphorylated STAT2-mediated recruitment of STAT1, which is then phosphorylated at Tyr-701. Active phosphorylated STAT1/STAT2 heterodimers are released into the cytosol and translocated to the nucleus with ISGF3G, to form interferon-stimulated genes factor-3 (ISGF3) heterotrimers [34]. ISGF3 binds IFNA sequence response elements (ISREs) in the promoters of target genes via the DNA-binding domains of STAT1 and ISGF3G [35].

In humans, recessive complete STAT1 deficiency results in impaired responses to both IFNG and IFNA [36,37]. It is associated with a specific syndrome, different from MSMD, of susceptibility to both mycobacteria (impaired IFNG-mediated immunity) and viruses (impaired IFNA-mediated immunity). The three known patients were unrelated and homozygous for specific loss-of-function and loss-of-expression *STAT1* mutations [36,37]. No STAT1 proteins were detected in the patients' cells and, accordingly, no activation of GAF and ISGF3 was found in response to IFNG and IFNA, respectively. The heterozygous relatives of these patients were healthy. This contrasts with the heterozygous L706S *STAT1* mutation described in three patients from two kindreds with dominant partial STAT1 deficiency and MSMD [17]. The L706S allele is null (loss-of-function) for the activation of both GAF and ISGF3, despite normal levels of STAT1 production. Intriguingly, this allele was found to be dominant for GAF activation but recessive for ISGF3 activation. This accounted for the patient's clinical phenotype of mycobacterial but not viral disease, but raised the question as to whether this genetic curiosity was of general relevance. We report here two novel *STAT1* alleles associated with MSMD: E320Q and Q463H. Like L706S, but by completely different mechanisms, these alleles are dominant for IFNG-induced GAF-mediated but recessive for IFNA-induced ISGF3-mediated immunity.

## Results

### Novel *STAT1* Mutations in Two Kindreds

**Figure 2:**
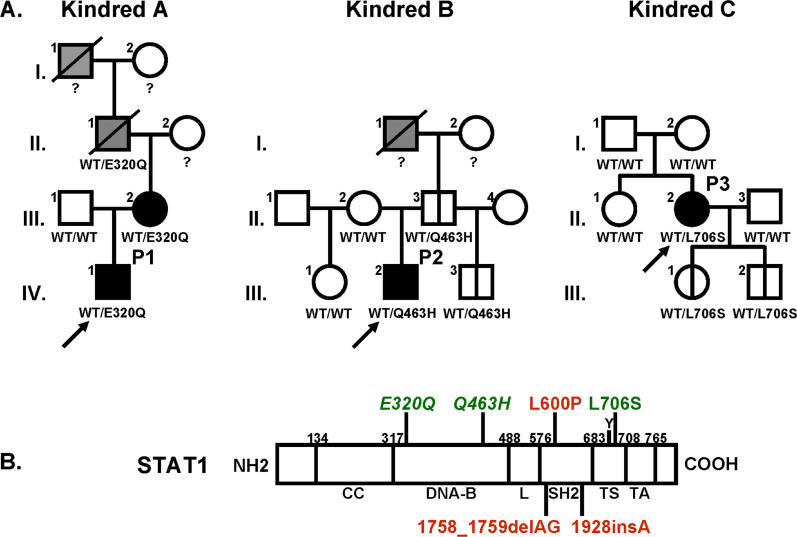
Novel *STAT1* Mutations in Two Kindreds (A) *STAT1* genotype and clinical phenotype of three kindreds. In kindred A, members I.1 and II.1 had tuberculosis, and III.2 and IV.1 (P1) had severe BCG disease. In kindred B, members I.1 and III. 2 (P2) were infected with M. tuberculosis and *M. avium,* respectively. Kindred C has been described elsewhere; II.2 (P3) developed disseminated BCG disease. Individuals with clinical disease caused by weakly virulent (BCG or M. avium) and more virulent *(M. tuberculosis)* mycobacteria are indicated in black and gray, respectively, and healthy individuals are shown in white. The index cases are indicated with an arrow. Genetically affected individuals (heterozygous for any of the three *STAT1* mutations) with no clinical phenotype at the time of this study are indicated by a vertical line. Known *STAT1* genotypes (WT, E320Q, Q463H, L706S) are indicated under each individual, with a question mark indicating unknown genotype. (B) The human *STAT1* coding region is shown, with its known pathogenic mutations. The coiled-coil domain (CC), DNA-binding domain (DNA-B), linker domain (L), SH2 domain (SH2), tail segment domain (TS), and *trans*-activator domain (TA) are indicated, together with their amino-acid boundaries. Tyrosine 701 (Y) is also indicated. Mutations in red are recessive mutations associated with complete STAT1 deficiency (due to a lack of STAT1 production), impaired IFNG-induced GAF activation and IFNA-induced ISGF3 activation, and a syndrome of predisposition to mycobacterial and severe viral disease in homozygous individuals. Mutations in green are associated, in heterozygous individuals, with partial STAT1 deficiency (normal STAT1 expression), impaired IFNG-induced GAS-binding activity but normal IFNA-induced ISRE-binding activity, and MSMD (predisposition to mycobacterial but not viral disease). Mutations reported for the first time in this study are indicated in italics.

We investigated two unrelated children (P1 and P2) with a mild form of MSMD (Figure 2A, case reports). The coding regions of the five genes known to be associated with MSMD *(IL12B, IL12RB1, IFNGR1, IFNGR2,* and *STAT1)* were sequenced following amplification of the corresponding cDNAs from Epstein-Barr virus (EBV)–transformed B cells from the patients. Mutations were found in *STAT1* cDNAs, and were confirmed by sequencing products amplified from the corresponding *STAT1* genomic exons in EBV-transformed B cells and fresh blood cells. No mutations were found in the coding regions of the other four genes. A heterozygous nucleotide substitution at position 958 (G→C) was found in *STAT1* exon 11 in P1, leading to a Glu→Gln substitution at position 320 (E320Q). A heterozygous nucleotide substitution at position 1389 (G→T) was found in exon 17 in P2, leading to a Gln→His substitution at position 463 (Q463H). These mutations were not found in 75 unrelated European control individuals tested (150 chromosomes), suggesting they are not merely irrelevant polymorphisms. Moreover, P1′s mother (A.III.2) had MSMD and was found to be heterozygous for the E320Q allele. Analysis of genomic DNA extracted from biopsy tissue taken from the deceased maternal grandfather of P1 (A.II.1), who had suffered from bona fide tuberculosis, showed that he too was heterozygous for the E320Q allele, providing further evidence for dominant cosegregation of the *STAT1* genotype and clinical phenotype. However, healthy relatives of P2 (B.II.3 and B.III.3) were found to be heterozygous for the Q463H mutation, suggesting that this allele (and possibly E320Q), like the previously reported L706S *STAT1* allele [17], is associated with a partial form of STAT1 deficiency, with low clinical penetrance.

### Molecular Representation of STAT1 Mutants

**Figure 3:**
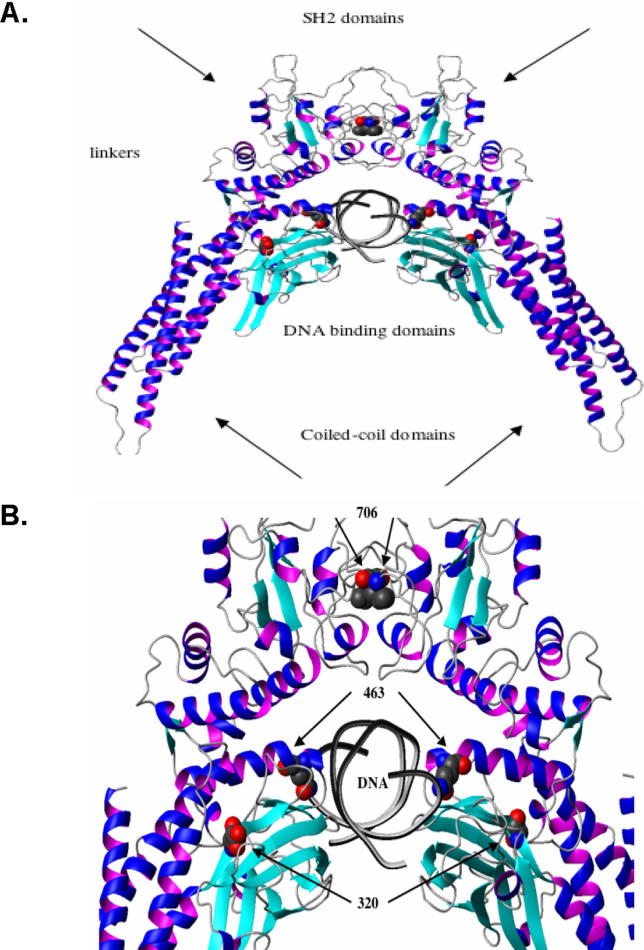
Molecular Representation of STAT1 Mutants (A) Ribbon representation of the WT STAT1 homodimer complexed with DNA. Secondary structure elements representing the β strands are shown in cyan, and the helices are shown in blue-magenta. Atoms of residues in mutated positions L706, E320, and Q463 (indicated by arrows) are shown in space-filling models. Atoms are shown in red (for oxygen), blue (for nitrogen), and gray (for carbon). (B) Magnified focus on the region containing the three mutated residues.

According to the crystallographic structure of phosphorylated STAT1 dimers bound to DNA, the E320Q and Q463H mutations affect residues from the DNA-binding domain of STAT1 that are conserved in all known human STAT molecules [32]. We therefore attempted to predict the effect of these mutations on the binding of STAT1 homodimers to their GAS target region. The 3D models of the mutants shed light on the potential impact of these mutations on the binding of STAT1 homodimers to their GAS target DNA element (Figure 3A, 3B, and unpublished data). The acidic E320 residue is located in a bulge at the very beginning of the eight-strand β-barrel (Figure 3). It helps to stabilize the structure through the salt bridge it forms with K344 and R346 on the adjacent antiparallel strand, which contains residues in direct contact with DNA. Residue Q463 is directly involved in DNA binding, forming a hydrogen bond with thymidine, and coming into contact with the phosphate backbone of DNA. It also forms hydrogen bonds with residues S459 and N460, thereby stabilizing a loop that contacts DNA. These in silico studies suggest that the E320Q and Q463H mutations in *STAT1* are deleterious, as they are expected to affect the GAS DNA-binding activity of the corresponding mutant STAT1 proteins either directly or indirectly. They also suggest that the E320Q allele may be less severe than the Q463H allele. In contrast, residue L706 is located in the C-terminal tail segment (residues 701–708), which is involved in STAT1 activation by phosphorylation of Y701 and STAT1 homodimerization. Residue L706 from one STAT1 molecule packs against a hydrophobic pocket formed by the side-chains of L706, I707, A641, and V642 from the other STAT1 molecule. The L706S mutation might therefore be expected to impair the Y701 phosphorylation and dimerization of STAT1.

### Normal Activation of STAT1 in Heterozygous Cells from Patients

**Figure 4:**
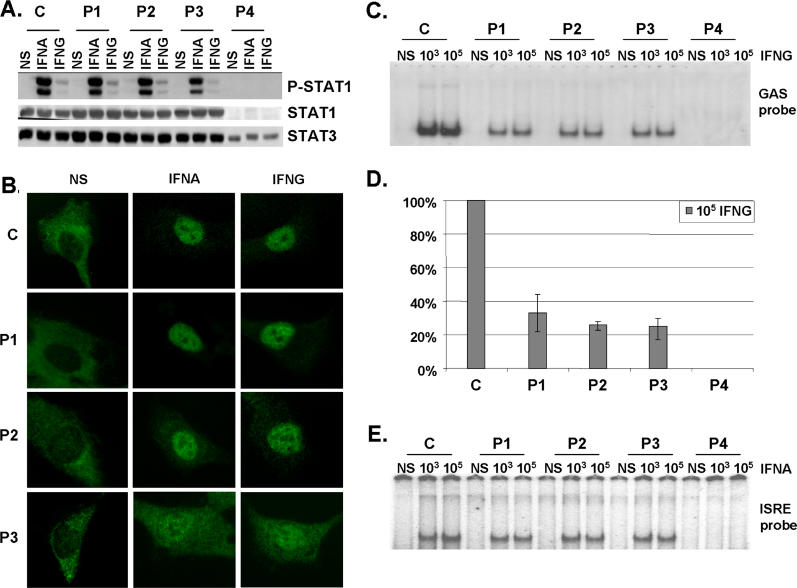
Normal Activation but Impaired DNA-Binding Activity of STAT1 in Heterozygous Cells from Patients (A) Western blot of total protein extracts (100 μg) from EBV-transformed B cells derived from a healthy control (C), three patients under study (P1, P2, P3), and a patient with recessive complete STAT1 deficiency (P4) homozygous for the 1758_1759delAG mutation, probed with specific antibodies against phosphorylated-Tyr-701-STAT1, STAT1, and STAT3. EBV-transformed B cells were not stimulated (NS) or were stimulated with IFNA or IFNG (10^5^ IU/ml) for 30 min. (B) Immunofluorescence staining with a STAT1-specific antibody of skin-derived SV40-transformed fibroblasts from a healthy control (C) and three patients under study (P1, P2, P3). Fibroblasts were not stimulated (NS) or were stimulated with IFNA or IFNG (10^5^ IU/ml) for 30 min. (C and E) EMSA of nuclear extracts (5 μg) from EBV-transformed B cells derived from a healthy control (C), three patients under study (P1, P2, P3), and the patient with complete STAT1 deficiency (P4). EBV-transformed B cells were not stimulated (NS) or were stimulated for 30 min with 10^3^ and 10^5^ IU/ml of IFNG (C) and IFNA (E), respectively. Radiolabeled GAS (C) or ISRE (E) probes were used. (D) Quantification of four to six independent experiments by PhosphoImager SI (Molecular Dynamics, Piscataway, New Jersey, United States) using the GAS probe in response to 10^5^ IU/ml of IFNG is also presented. The mean, minimum, and maximum values are expressed with respect to the positive control response (100%). For (A–C) and (E), one experiment representative of three to five independent experiments is shown.

We assessed STAT1 production in EBV-transformed B cells from a healthy control individual (C), the patients under study (P1 and P2), a previously reported patient with partial STAT1 deficiency carrying the heterozygous L706S *STAT1* mutation (P3) [17], and a patient with complete STAT1 deficiency carrying the homozygous 1758_1759delAG *STAT1* frameshift deletion (P4) [36] by Western blotting with a specific antibody (Figure 4A). Cells from P1, P2, and P3 produced similar amounts of STAT1 protein to control cells (C), whereas STAT1 was absent in cells from P4 (Figure 4A and Table 2). Cells from P3 contained about half the normal amount of Y701-phosphorylated STAT1 following treatment with either type of IFN (Figure 4A), as previously described [17]. In contrast, cells from P1 and P2 had normal levels of Y701-phosphorylated STAT1 following treatment with either type of IFN (Figure 4A and Table 2). We then investigated the nuclear translocation of STAT1 upon IFN stimulation in simian virus 40 (SV40)–transformed fibroblasts from a healthy control (C), patients P1, P2, and P3, the STAT1-deficient fibrosarcoma cell line U3C, and its parental cell line 2C4 by immunofluorescence staining with a specific antibody. STAT1 nuclear accumulation in response to both IFNA and IFNG was normal in fibroblasts from P1 and P2, whereas it was, as expected, impaired in cells from P3 (Figure 4B and Table 2) [17]. No staining was observed in U3C STAT1-deficient fibroblasts incubated with a STAT1-specific antibody, or in any other fibroblast line incubated with an isotypic control antibody (unpublished data). Staining with a STAT2-specific antibody indicated that the STAT1-containing complexes accumulating in the nucleus in response to IFNA consisted, at least partly, of ISGF3 ([17,36] and unpublished data). Thus, the E320Q and Q463H *STAT1* mutations do not seem to impair the production of STAT1, its phosphorylation at Y701 (activation), or its accumulation in the nucleus following the stimulation of heterozygous cells with IFNA and IFNG.

**Table 2:**
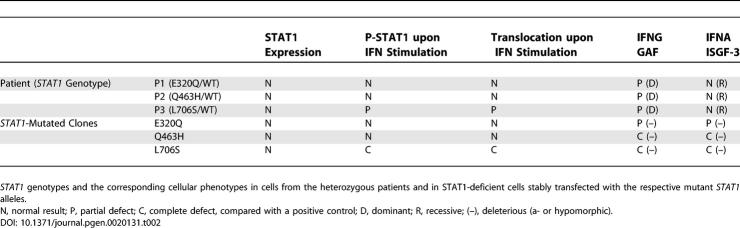
*STAT1* Genotypes and Associated Cellular Phenotypes

### Impaired STAT1 DNA-Binding Activity in Heterozygous Cells from the Patients

We further investigated the impact of the mutations by assessing the DNA-binding activity of GAF and ISGF3 in EBV-transformed B cells stimulated with IFNG and IFNA by electrophoretic mobility shift assay (EMSA; Figure 4C–4E). Upon IFNG stimulation, cells from P1, P2, and P3 showed impaired GAS-binding activity (Figure 4C, 4D, and Table 2). The GAS-binding activity detected in cells from P2 and P3 was found to be about 25% of normal levels following treatment with 10^5^ IU/ml IFNG (mean of 4 experiments, ± 5%) [17] (Figure 4D). The GAS-binding activity detected in cells from P1 was found to be mildly, but reproducibly higher, at about 33% (mean of 6 experiments, ± 9%) (Figure 4D). Seventeen controls were tested and variation was found to be less than 20% of the mean value (unpublished data). The GAS-binding activity of P1, P2, and P3 on IFNA stimulation was also found to be impaired, indicating that the phenotype observed was strictly STAT1 dependent and independent of the amount of phosphorylated STAT1 (Figure S1A). Upon IFNA stimulation, P1, P2, and P3 presented normal IFN-stimulated response element (ISRE)–binding activity [17] (Figure 4E and Table 2). The addition of unlabeled probes to the EMSA indicated that the signals detected were GAS and ISRE specific (unpublished data). Supershift experiments with STAT1-, STAT2-, STAT3-, and ISGF3G-specific antibodies indicated that GAS- and ISRE-binding activities were mediated by GAF and ISGF3, respectively (unpublished data). Finally, we observed strict cosegregation of the *STAT1* genotype and cellular phenotype, with impaired IFNG-induced GAS-binding activity in all individuals heterozygous for a mutant *STAT1* allele, and in none of their wild-type homozygous relatives (data not shown). These data suggest that the E320Q and Q463H *STAT1* alleles are pathogenic and associated with dominant and partial STAT1 deficiency, and with MSMD of low clinical penetrance, due to impaired GAS binding by nuclear phophorylated STAT1 homodimers in response to IFNG. We cannot discriminate whether mutated proteins bind with less or no affinity or bind with a greater off rate.

### Normal Activation of STAT1 Mutants in Stable Transfectants

**Figure 5:**
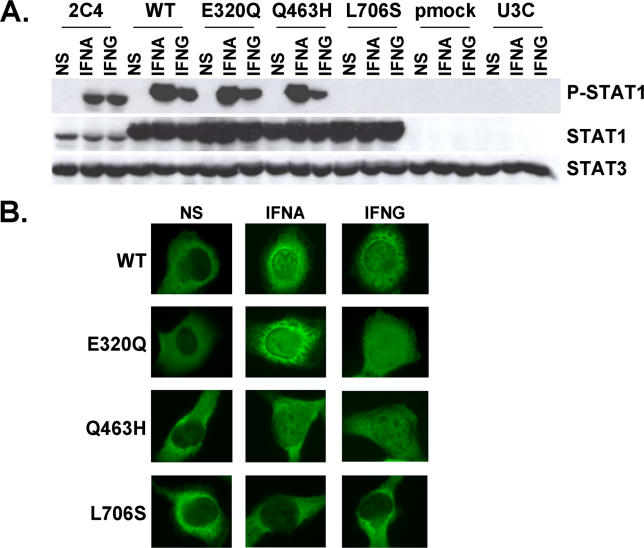
Normal Activation of STAT1 Mutants in Stable Transfectants (A) Western blot of total protein extracts (100 μg) from a parental fibrosarcoma cell line (2C4) and STAT1-deficient U3C fibrosarcoma cell clones, untransfected (U3C) or stably cotransfected with a zeocin-resistance vector and a vector containing a mock (pmock), WT, E320Q, Q463H, or L706S *STAT1* allele, with antibodies specific for phosphorylated-Tyr-701-STAT1, STAT1, and STAT3. The cells were not stimulated (NS) or were stimulated for 30 min with 10^5^ IU/ml IFNA or IFNG. (B) Immunofluorescence staining, with a STAT1-specific antibody, of STAT1-deficient U3C fibrosarcoma cell clones, stably cotransfected with a zeocin-resistance vector and a vector containing a WT, E320Q, Q463H, or L706S *STAT1* allele. The cells were not stimulated (NS) or were stimulated with IFNA or IFNG (10^5^ IU/ml) for 30 min. For (A) and (B), one experiment representative of three independent experiments is shown.

Experiments conducted in heterozygous cells do not allow precise dissection of the molecular mechanisms involved. We therefore cotransfected the STAT1-deficient U3C fibroblast line with a zeocin-resistance vector and a vector carrying mock or *STAT1* alleles (wild-type [WT], E320Q, Q463H, and L706S), and selected stable clones on the basis of zeocin resistance. We assessed STAT1 production in the various cell lines by Western blotting with a specific antibody (Figure 5A). The WT, E320Q, Q463H, and L706S alleles were overexpressed with respect to the control cell line (2C4). No STAT1 was detected in parental U3C cells or stable clones transfected with a mock vector. STAT1 was normally phosphorylated at Y701 upon stimulation with either IFNA or IFNG in the WT, E320Q, and Q463H cell lines, but not in the L706S cell line (Figure 5A and Table 2). We then assessed the nuclear accumulation of STAT1 upon stimulation with IFNA and IFNG by immunofluorescence assays with a specific antibody (Figure 5B). Nuclear accumulation was observed for WT, E320Q, and Q463H STAT1, but not for L706S STAT1 molecules, in response to both IFNG and IFNA (Figure 5B and Table 2). No fluorescence was observed in U3C parental cells or stable clones transfected with a mock vector or using an isotypic control antibody (unpublished data). These data confirm that the *STAT1* L706S mutation is associated with a complete lack of Y701 phosphorylation and subsequent nuclear translocation of STAT1 upon stimulation with IFNA and IFNG [17]. Our findings also indicate that the *STAT1* E320Q and Q463H alleles encode proteins that are normally phosphorylated at Y701 and translocated upon stimulation with either IFNA or IFNG [31].

### Impaired DNA-Binding Activity of STAT1 Mutants in Stable Transfectants

**Figure 6:**
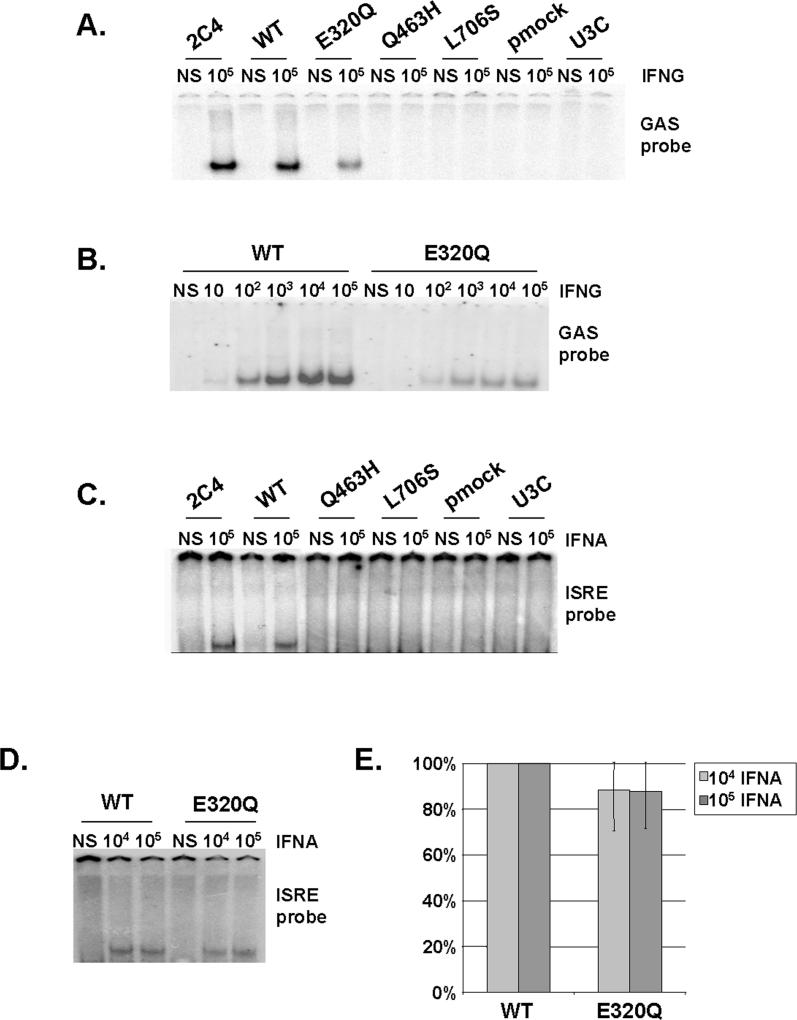
Impaired DNA-Binding Activity of STAT1 Mutants in Stable Transfectants (A–D) EMSA of nuclear extracts (5 μg [A and B]; 30 μg [C and D]) from a parental fibrosarcoma cell line (2C4) and STAT1-deficient U3C fibrosarcoma cell clones, untransfected (U3C) or stably cotransfected with a zeocin-resistance vector and a vector containing a mock (pmock), WT, E320Q, Q463H, or L706S *STAT1* allele. The cells were not stimulated (NS) or were stimulated for 30 min with the indicated doses of IFNG (A and B) or IFNA (C and D). We used the radiolabeled GAS probe FCGR1 (A and B) or an ISRE probe (C and D). For (A–D), one experiment representative of three to five independent experiments is shown. (E) Quantification of three independent experiments by PhosphoImager SI (Molecular Dynamics), using the ISRE probe, in response to 10^4^ and 10^5^ IU/ml IFNA is also presented. The mean, minimum, and maximum values are expressed with respect to the WT stable transfectant clone response (100%).

We then assessed the impact of *STAT1* mutations on DNA-binding activity in EMSA. In response to even high doses of IFNG (10^5^ IU/ml), no GAS binding was detected with the Q463H and L706S *STAT1* alleles, despite the use of several GAS probes (FCGR1, M67, and IRF1) (Figure 6A; unpublished data and Table 2). E320Q cell lines (producing similar amounts of STAT1 to the WT; Figure 6A and Table 2) were found to have impaired, but not abolished GAS-binding activity at various concentrations of IFNG (Figure 6A and 6B) (37%, mean of five experiments, ±7%). Similar results were obtained upon IFNA stimulation, indicating the phenotype observed was strictly STAT1 dependent and independent of the amount of phosphorylated STAT1 (Figure S1B and S1C). In addition, Q463H and L706S were found to be associated with a lack of ISRE-binding activity on stimulation with even high doses of IFNA (10^5^ IU/ml) (Figure 6C and Table 2), whereas the E320Q allele displayed only mildly impaired binding (90%, mean of three experiments, ± 17%) at the two highest concentrations of IFNA (Figure 6D, 6E, and Table 2). This reflects the known involvement of STAT1 with ISGF3G in ISGF3 binding to ISRE [35], and indicates that Q463H and, to a lesser extent, E320Q, impair this process. The GAS- and ISRE-binding activities of WT and E320Q cells were shown to involve GAF and ISGF3, respectively, in competition experiments with unlabeled probe and supershift experiments (unpublished data). Thus, the L706S *STAT1* allele is therefore null for the DNA-binding activity of both GAF (to GAS elements) and ISGF3 (to ISRE elements), as previously shown in transient transfectants [17]. This defect is due to impaired Y701 phosphorylation and nuclear accumulation. The Q463H allele is also null for both cellular phenotypes, but the underlying mechanism is different, involving impaired DNA binding by nuclear STAT1-containing complexes. The E320Q allele acts by a similar mechanism, affecting DNA binding, but has a milder impact, being markedly hypomorphic for GAS-binding activity and barely hypomorphic for ISRE-binding activity. We cannot determine whether mutated proteins bind with less or no affinity or bind with a greater off rate.

### Impact of *STAT1* Mutations on Transcription

**Figure 7:**
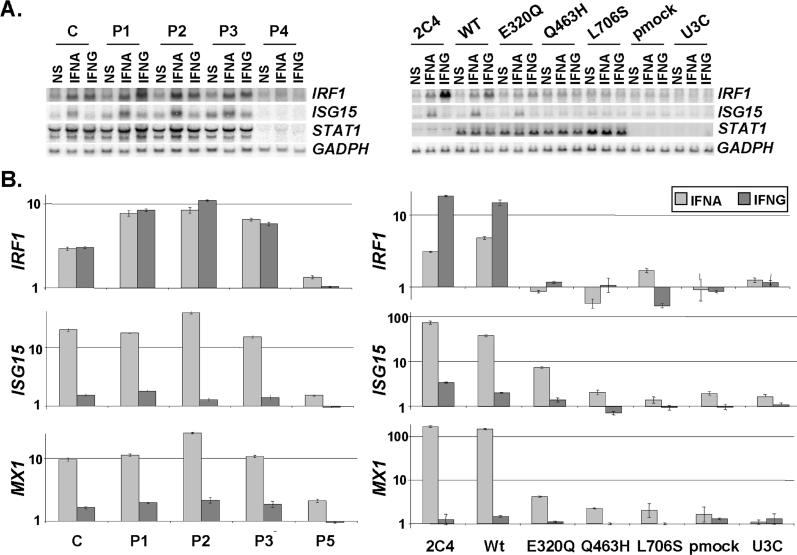
Impact of *STAT1* Mutations on Transcription (A) Levels of mRNA corresponding to *STAT1,* IFNG- and/or IFNA-inducible genes *(IRF1* and *ISG15)* and *GADP* in EBV-transformed B cells from a control (C), the three affected individuals (P1, P2, and P3) and a STAT1-deficient individual (P4), or in the 2C4 parental fibrosarcoma cell line, STAT1-deficient U3C cell line, and U3C cells stably transfected with a mock (pmock), WT, E320Q, Q463H, or L706S *STAT1* allele, either not stimulated (NS), or stimulated for 2 h with 10^3^ of IFNG or 10^4^ of IFNA for EBV-transformed B cells and 10^5^ IU/ml of IFNG or IFNA for fibroblasts, as detected by Northern blotting. (B) Relative real-time PCR of *IRF1, ISG15,* and *MX1,* and cDNAs from EBV-transformed B cells derived from a healthy control (C), three patients under study (P1, P2, P3), and a patient with recessive complete STAT1 deficiency (P5) homozygous for the 1928insA *STAT1* mutation or from parental fibrosarcoma cell line (2C4) and STAT1-deficient U3C fibrosarcoma cell clones, untransfected (U3C) or stably cotransfected with a zeocin-resistance vector and a vector containing a mock, WT, E320Q, Q463H, or L706S *STAT1* allele stimulated or not stimulated with 10^5^ IU/ml of IFNG or IFNA for 1 h and 2 h for EBV-transformed B cells and fibroblasts, respectively, for *IRF1,* and for 6 h for both cellular types for *ISG15* and *MX1*. Means values of duplicates of one experiment are shown with their respective standard variations.

We validated our findings by assessing the impact of *STAT1* mutations on transcription by Northern blotting and relative real-time RT-PCR to determine the amount of mRNA for various IFNG- and IFNA-inducible genes [36] in EBV-transformed B cells from the patients (Figure 7A and 7B). Target genes *ISG15* (the promoter of which was used for EMSA), *MX1,* and *IRF1* (the promoter of which was used for EMSA) were induced following stimulation with IFNA and IFNG, as appropriate, in control cells, but not in cells from a patient lacking STAT1 (P5, homozygous for the 1928insA *STAT1* allele, which encodes no detectable protein) [37]. Cells from heterozygous patients P1, P2, and P3 showed no impairment of the induction of STAT1-dependent target genes in response to IFNG and IFNA. This finding confirms that these patients suffer from partial, as opposed to complete, STAT1 deficiency, with a mild effect on GAF-mediated IFNG responses and not on ISGF3-mediated IFNA responses [17,36]. We then assessed the induction of the same target genes in stably transfected fibroblasts (Figure 7A and 7B). Target genes were induced in control cells and in stable clones transfected with WT *STAT1* allele, but not in STAT1-deficient U3C and in stable clones transfected with a mock vector. They were not induced in either L706S or Q463H transfectants. This confirms that the L706S and Q463H alleles are loss-of-function alleles for IFNG-driven GAF and IFNA-driven ISGF3 activation. In contrast, the induction of *ISG15* and *MX1* were impaired, but not abolished in cells expressing the E320Q hypomorphic allele. The induction of *IRF1* was abolished. These data confirm that (1) the three mutant *STAT1* alleles are associated with partial, as opposed to complete STAT1 deficiency for GAF-dependent IFNG-inducible genes in heterozygous patients; and (2) the L706S and Q463H alleles are intrinsically loss-of-function, whereas the E320Q allele is hypomorphic for both GAF-dependent IFNG-inducible and ISGF3-dependent IFNA-inducible gene transcription.

### Mechanism of Dominance of the *STAT1* Alleles for GAS-Binding Activity

**Figure 8:**
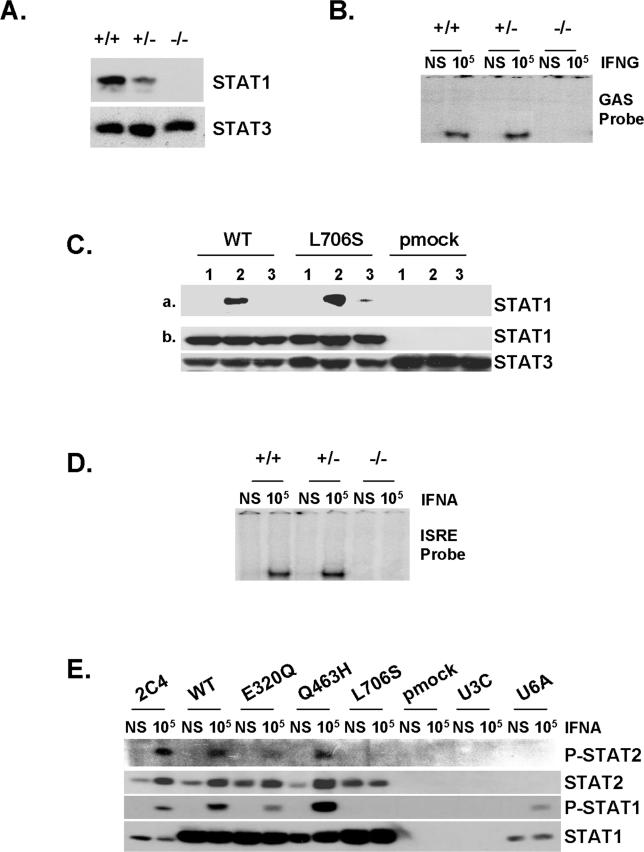
Mechanism of Dominance of the *STAT1* Alleles for GAS-Binding Activity and of Recessivity of the *STAT1* Alleles for ISRE-Binding Activity (A) Western blot of total protein extracts (100 μg) from unstimulated EBV-transformed B-cell lines from a healthy control (+/+), P4′s father (+/−) (heterozygous for the loss-of-expression, loss-of-function *STAT1* 1758_1759delAG allele), and P5 (−/−) (homozygous for the loss-of-expression, loss-of-function *STAT1* 1928insA allele), using antibodies specific for STAT1 and STAT3. (B) EMSA of nuclear extracts (5 μg) from EBV-transformed B cells derived from a healthy control (+/+), P5 (−/−) (homozygous for the loss-of-expression, loss-of-function *STAT1* 1928insA allele), and P4′s father (+/−) (heterozygous for the loss-of-expression, loss-of-function *STAT1* 1758_1759delAG allele). EBV-transformed B cells were not stimulated (NS) or were stimulated for 30 min with 10^5^ IU/ml IFNG. A radiolabeled GAS (FCGR1) probe was used. (C) (a) Whole-cell extracts of 10^7^ STAT1-deficient U3C fibrosarcoma cell clones stably cotransfected with a zeocin-resistance vector and a vector containing a mock (pmock), WT, or L706S *STAT1* allele were subjected to immunoprecipitation with the following biotinylated peptides: TSFGYDKPHVLV (1), corresponding to the intracellular part of IFNGR1 around the unphosphorylated Tyr-440 residue (Y); TSFG(pTyr)DKPHVLV (2), corresponding to the intracellular part of IFNGR1 around the phosphorylated Tyr-440 residue (pTyr); and SLIG(pTyr)RPTEDSK (3), corresponding to an irrelevant peptide similar to peptide 2. (b) 20 μL of each extract was taken before immunoprecipitation, and Western blotting was performed with STAT1- and STAT3-specific antibodies. (D) EMSA of nuclear extracts (5 μg) from EBV-transformed B cells derived from a healthy control (C), P4′s father (+/−) (heterozygous for the loss-of-expression, loss-of-function *STAT1* 1758_1759delAG allele) and P5 (−/−) (homozygous for the loss-of-expression, loss-of-function *STAT1* 1928insA allele). EBV-transformed B cells were not stimulated (NS) or were stimulated for 30 min with 10^5^ IU/ml of IFNA. A radiolabeled ISRE probe was used. (E) Immunoprecipitation with a STAT1-specific antibody, followed by Western blotting with Tyr701-phospho-STAT1–specific, Tyr690-phospho-STAT2–specific, STAT1-specific, and STAT2-specific antibodies, of total protein extracts (1 mg) from a parental fibrosarcoma cell line (2C4), a STAT2-deficient U6A fibrosarcoma cell line, and STAT1-deficient U3C fibrosarcoma cell clones, untransfected (U3C) or stably cotransfected with a zeocin-resistance vector and a vector containing a mock, WT, E320Q, Q463H, or L706S *STAT1* allele. The cells were not stimulated (NS) or were stimulated for 30 min with 10^5^ IU/ml IFNA. For (B–E), one experiment representative of two independent experiments is shown.

We investigated the molecular mechanisms of dominance of E320Q, Q463H, and L706S *STAT1* alleles by studying EBV-transformed B cells from P4′s father (+/−) (heterozygous for the 1758_1759delAG *STAT1* allele encoding no detectable STAT1 [36]). These cells produced half the amount of STAT1 detected in six control cell lines (Figure 8A; unpublished data) but responded normally to IFNG in terms of GAS-binding activity (Figure 8B). This clearly demonstrates that *STAT1* mutant alleles cannot be dominant due to haploinsufficiency, implying the involvement of a negative-dominance mechanism for the three mutant *STAT1* alleles studied here. We previously dissected the molecular mechanism involved in the loss-of-function and dominance associated with the L706S allele [17]. The docking site of unphosphorylated STAT1 on IFNGR1 has been shown to be created by the phosphorylation of Tyr 440 upon IFNG stimulation [30]. We further dissected this molecular mechanism underlying the negative dominance of the L706S allele, using biotinylated peptides corresponding to an intracellular segment of IFNGR1, unphosphorylated or phosphorylated at Tyr 440, or an irrelevant phosphorylated peptide. We incubated the peptides with whole-cell protein extracts from WT, L706S, and mock stable transfectant cell lines. Streptavidin immunoprecipitates were analyzed by Western blotting with a STAT1-specific antibody (Figure 8C). We found that the phosphorylated IFNGR1 peptide, unlike the other two peptides, interacted strongly with WT and L706S STAT1 molecules, indicating that the L706S mutation does not impair the recruitment of STAT1 by phosphorylated IFNGR1 upon IFNG stimulation. The loss-of-phosphorylation associated with the L706S allele is therefore necessary and sufficient to account for the associated loss-of-function and the dominance of this allele. The loss-of-function Q463H allele, acting by a different mechanism, is also dominant for GAF activation in response to IFNG: only one in four combinations of STAT1-activated phosphodimers binds GAS correctly in the Q463H/WT cells from P2 (25% of normal levels). Interestingly, E320Q/WT cells from P1 had a higher level of IFNG-induced GAS-binding activity (33% of normal levels). The E320Q allele is dominant, for the same reasons as Q463H, but gives stronger responses to IFNG because it is hypomorphic (some E320Q-containing GAF molecules do bind GAS; Figure 6A and 6B).

### Mechanism of Recessivity of *STAT1* Alleles for ISRE-Binding Activity

We investigated the molecular mechanisms underlying the recessivity of the three deleterious mutations E320Q, Q463H, and L706S for ISGF3 binding to ISRE. We investigated P4′s father, who produces half as much STAT1 protein as the control (Figure 8A). His EBV-transformed B cells activated normal amounts of ISRE-binding ISGF3 proteins upon stimulation with IFNA (Figure 8D). Thus, deleterious *STAT1* alleles, whether affecting Y701 phosphorylation or DNA binding, cannot be haploinsufficient for ISGF3 binding to ISRE. Moreover, as ISGF3 complexes contain a single STAT1 molecule bound to STAT2 and ISGF3G (unlike GAF, which contains two STAT1 molecules), a mechanism involving the negative dominance of mutant *STAT1* alleles would require mutant STAT1 proteins to bind preferentially to STAT2 and ISGF3G, increasing the proportion of inactive ISGF3 complexes. We thus investigated whether the E320Q, Q463H, and L706S STAT1 proteins could interact with nonphosphorylated STAT2 in unstimulated cells (preassociation [29,38,39]), and with tyrosine-phosphorylated STAT2 upon IFNA stimulation [40]. We subjected stably transfected fibroblast cell lines, left unstimulated or stimulated with IFNA, to immunoprecipitation with a STAT1-specific antibody, followed by Western blotting with specific antibodies directed against tyrosine-phosphorylated STAT2, STAT2, tyrosine-phosphorylated STAT1, and STAT1 (Figure 8E). Similar levels of preassociation between nonphosphorylated WT, E320Q, Q463H, and L706S STAT1 molecules and nonphosphorylated STAT2 were observed in resting cells. Upon IFNA stimulation, phosphorylated WT, E320Q, and Q463H STAT1 molecules interacted equally strongly with phosphorylated STAT2, whereas L706S displayed no high-affinity interaction with phosphorylated STAT2. This may reflect the documented lack of L706S phosphorylation at Y701, or the predicted impairment of Stat phosphodimer formation due to replacement of the key residue L706 [32], or both. Thus, (1) nonphosphorylated L706S STAT1 molecules are not associated with phosphorylated STAT2 upon IFNA stimulation, revealing a second impact of L706S, in addition to the loss of Y701 phosphorylation, accounting in part for the recessive nature of this mutation for ISGF3 activation; and (2) the E320Q and Q463H STAT1 mutants are normally recruited by phosphorylated STAT2, with which they form phosphorylated heterodimers. All three alleles are therefore recessive because no more than half of the ISGF3 complexes (none in the case of L706S) contain a mutant STAT1 molecule in heterozygous cells, and there is no haploinsufficiency.

### Impact of Mutant *STAT1* Alleles on IFNG-Mediated Immunity

**Figure 9:**
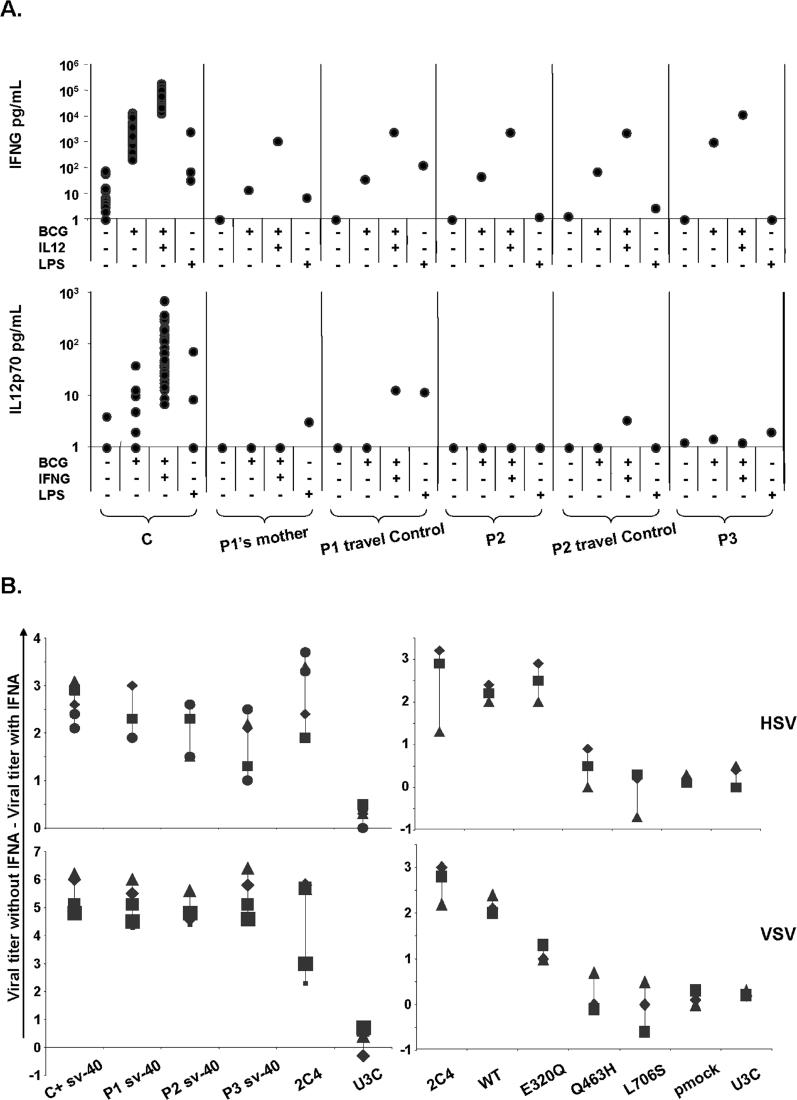
Impact of Mutant *STAT1* Alleles on IFNG- and IFNA-Mediated Immunity (A) Cytokine production in the supernatant of whole blood from healthy controls (C) and patients (P1′s mother, P2, and P3) and their respective “travel” control, not stimulated (NS) or stimulated for 72 h with live BCG alone or BCG plus IL12 or IFNG. The levels of IFNG and IL12 in the supernatant were determined by enzyme-linked immunosorbent assay. One experiment representative of two independent experiments is shown. (B) Skin-derived SV40-transformed fibroblasts from a healthy control (C), the three patients under study (P1, P2, P3), a parental fibrosarcoma cell line (2C4), STAT1-deficient U3C fibrosarcoma cell line (U3C), and U3C clones stably transfected with a mock (pmock), WT, E320Q, Q463H, or L706S *STAT1* alleles, were infected with HSV-1 or VSV, with or without priorstimulation with IFNA (10^5^ IU/ml) for 24 h. Viral titers were determined after 48 h of infection. Five independent experiments are shown for the patient's cells and three independent experiments are shown for sarcoma fibroblasts. Each assay is symbolized by a different character.

We evaluated the physiological relevance of our studies by assessing the impact of the E320Q, Q463H, and L706S *STAT1* alleles on IFNG-dependent immunity in a whole-blood assay by studying the late induction of IL12p70 in response to live bacille Calmette-Guérin (BCG), or live BCG plus IFNG, and the induction of IFNG production in response to live BCG, or live BCG plus IL12, in the patients [41] (Figure 9A). We studied blood cells from P1′s mother, rather than from P1, for convenience. These cells had the same *STAT1* genotype and cellular phenotype as the cells of P1 in terms of the response to IFNG of EBV-transformed B cells. Accordingly, the response to BCG plus IL12 was normal in terms of IFNG production. Moreover, P1′s mother, P2, and P3 presented a complete lack of induction of IL12p70 in response to BCG plus IFNG (Figure 9A), as in all patients with complete or partial IFNGR1, and IFNGR2 deficiencies [41]. IL12 production in response to LPS was studied as a control and was found to be normal in P1′s mother and P3. These immunological results, obtained with fresh blood cells, correlate with biochemical assays performed on the patient's EBV-transformed B cells. This cellular phenotype accounts for the immunological phenotype of impairment of the IL12–IFNG circuit upon stimulation of the blood cells with mycobacteria and IFNG ex vivo. This immunological phenotype is, in turn, correlated with the clinical phenotype of impaired antimycobacterial immunity in vivo.

### Impact of Mutant *STAT1* Alleles on IFNA-Mediated Immunity

We then evaluated the physiological relevance of our studies by assessing the impact of the E320Q, Q463H, and L706S *STAT1* alleles on IFNA-dependent immunity. We evaluated the response to IFNA of SV40-transformed fibroblasts from the patients, in terms of the control of herpes simplex virus (HSV) and vesicular stomatitis virus (VSV) infections (Figure 9B) [36,37]. Heterozygous cells from the patients controlled the growth of HSV and VSV normally in response to IFNA, consistent with the E320Q, Q463H, and L706S alleles being recessive for ISGF3 activation. We subjected stably transfected fibroblast cell lines to the same assay, and in contrast, cell lines expressing only Q463H or L706S controlled the growth of neither HSV nor VSV in response to IFNA, consistent with these alleles being null for ISGF3 activation (Figure 9B). The E320Q cell line presented no detectable defect in the control of HSV, but control of the more virulent VSV was clearly impaired. Similar data were reproducibly obtained with several concentrations of IFNA (data not shown). The deleterious impact of the hypomorphic E320Q allele therefore impaired, but did not completely abolish the response to IFNA, even at high doses (10^5^ IU/ml). These functional results are therefore correlated with the ISRE-binding and ISGF3 transcriptional phenotypes. Heterozygous cell lines displayed normal ISRE-binding activity of ISGF3 and the normal induction of target genes in response to IFNA. This biochemical phenotype accounts for the immunological phenotype of normal control of HSV and VSV growth in vitro. This immunological phenotype is correlated with a clinical phenotype of normal resistance to most viruses in vivo [17] (case reports). Thus, the deleterious E320Q, Q463H, and L706S mutations are dominant for GAS-binding and GAF transcriptional activity and antimycobacterial immunity and recessive for ISRE-binding and ISGF3 transcriptional activity and antiviral immunity.

## Discussion

We describe here a novel form of partial STAT1 deficiency, the 12th genetic etiology of MSMD to be identified and the second form of STAT1 deficiency shown to be associated with this syndrome [14,17]. The previously described L706S *STAT1* mutation in two kindreds leads to a defect in STAT1 phosphorylation at Y701 [17]. The E320Q and Q463H alleles result in impaired GAS binding by normally phosphorylated nuclear STAT1 homodimers. This finding has important clinical implications, because the screening of patients with MSMD by the detection of phosphorylated STAT1 in IFNG-stimulated blood cells would not detect this new form of STAT1 deficiency [42]. Measuring the induction of IL12 in response to IFNG is more appropriate as a first diagnostic step [41]. The clinical phenotype of patients with partial STAT1 deficiency is mild and resembles that of patients with partial IFNGR1 [19] and IFNGR2 [16] deficiencies. The penetrance of partial STAT1 deficiency is low, as only five of ten heterozygous patients from four kindreds [17] had case-definition infectious diseases caused by poorly virulent mycobacteria, reflecting the complex interplay between host and environmental factors in the course of infections [43,44]. Interestingly, one deceased patient (A.II.1) heterozygous for the E320Q allele suffered from bona fide tuberculosis. Two deceased relatives (A.I.1 and B.I.1) also had tuberculosis, possibly due to heterozygosity for the deleterious *STAT1* alleles. This finding is reminiscent of our observation of patients with IL12RB1 deficiency presenting with tuberculosis as their sole clinical phenotype [44,45].

The three mutant alleles are intrinsically deleterious for both IFNG-induced GAF-mediated and IFNA-induced ISGF3-mediated cellular activations, as shown in STAT1-deficient cells transfected with the corresponding alleles (Table 2 and Figure 1). There is no haploinsufficiency of *STAT1* for IFNG-induced GAF activation, and these three alleles are dominant for this pathway by negative interaction. L706S STAT1 is recruited to IFNGR1 but is not phosphorylated in response to IFNG. Consistent with the ordered, affinity-driven IFNG signaling process, only one (WT/WT) of the four possible STAT1 (WT/WT, WT/L706S, L706S/WT, L706S/L706S) combinations therefore forms phosphodimers in heterozygous cells. Whereas L706S exerts its negative effect in the cytosol, the other two mutations, E320Q and Q463H STAT1, exert their negative effects in the nucleus. Only one (WT/WT) of the four possible STAT1 combinations forms phosphodimers binding GAS elements with normal affinity. In contrast, all three alleles are recessive for the IFNA-induced ISGF3-mediated signaling pathway. There is neither haploinsufficiency nor negative interaction. The lack of negative dominance, in particular, is accounted for by the three STAT1 mutant proteins being no better recruited than WT STAT1 proteins to phospho-STAT2 and the IFNAR. Indeed, L706S STAT1 may not be recruited at all to IFNAR-bound phospho-STAT2. Altogether, the *STAT1* alleles studied here are dominant for IFNG-induced GAF-mediated signaling and recessive for IFNA-induced ISGF3-mediated signaling.

The three *STAT1* mutations are deleterious via different mechanisms, and they affect both cellular and clinical phenotypes, with a well-defined causal relationship between cellular defects and clinical diseases. Impaired IFNG-mediated immunity is associated with mycobacterial diseases, whereas impaired IFNA-mediated immunity is associated with viral diseases. It is widely accepted that the definition of candidate genes should take into account the observation that mutations in genes involved in multiple pathways in vitro can have an impact on only a few biochemical pathways in vivo, reflecting variable redundancy of the gene product. For example, complete STAT1 deficiency principally affects IFNA and IFNG responses in mice [46,47] and humans [36,37] in vivo, but STAT1 is involved, albeit in a redundant manner, in many other pathways studied in vitro. Our study shows that the definition of candidate genes, for both cellular and clinical phenotypes, should also take into account the possibility that the impact of the gene may depend on whether the organism studied is heterozygous or homozygous for the mutation. As a proof of principle, the three *STAT1* alleles profoundly affect IFNG-mediated immune responses to mycobacteria, but not IFNA-mediated immune responses to viruses, in heterozygous cells and patients, whereas they would affect both responses in homozygous cells and patients [36,37].

## Materials and Methods

### Patients.

P1 was born in Germany to unrelated German parents (Figure 2). He was vaccinated with BCG at birth. During the first year of his life, he developed disseminated BCG disease (BCG-osis) with fever, tuberculoid granulomatous inflammation, several small spots of osteolysis in his shoulder blade (close to the point at which the vaccine was administered), and a slightly enlarged spleen and enlarged lymph nodes in the lower abdomen. All conventional immunological assays (B cells, T cells, phagocytic respiratory burst) were normal. The patient was treated with antibiotics (isoniazid and rifampicin) for 7 mo and recovered. He was healthy at the age of 8 y as of June 2005. His mother developed local BCG disease (BCG-itis) after BCG vaccination at 14 y of age. She was healthy at the age of 38 y as of June 2005. P1′s maternal grandfather developed disseminated tuberculosis at the age of 16 y. At the age of 32 y, he developed a severe skin disease with deep lupus vulgaris-like ulcers that destroyed part of his nose. He died at the age of 49 y from liver cancer due to cirrhosis of unknown origin. P1′s mother's paternal grandfather died at the age of 38 y from tuberculosis. P1 had high titers of serum antibodies against mumps virus, varicella zoster virus, EBV, measles virus, rubella virus, influenza A virus, adenovirus, and respiratory syncytial virus. His mother had high titers of serum antibodies against hepatitis A virus, hepatitis B virus, varicella zoster virus, EBV, mumps virus, parvovirus B19, paranfluenza virus, adenovirus, respiratory syncytial virus, and enterovirus. The medical history of P2, also born to unrelated German parents in Germany, has been described elsewhere [48]. Briefly, he developed pulmonary M. avium infection at the age of 2 y. He was 10 y old as of June 2005 and well. P2 had high titers of serum antibodies against EBV, respiratory syncytial virus, adenovirus, parainfluenza virus (I and III), and influenza virus (A and B). His father was hospitalized at the age of 25 y for a fever of unknown origin. He recovered on antibiotic treatment and was 39 y old as of June 2005 and well. He had been vaccinated with BCG in infancy, with no adverse effect. P2′s paternal grandfather developed tuberculosis at the age of 20 y and recovered only after 5 y of hospitalization and antibiotic treatment in the 1950s. He died at the age of 50 y in unknown circumstances. P2′s half-brother has remained healthy and was 2.5 y old as of June 2005. The medical histories of P3, P4, and P5 have been described elsewhere [17,36,37]. P3 is heterozygous for the L706S *STAT1* mutation and developed BCG-osis after vaccination in childhood, but recovered after specific antibiotic therapy. P4 was homozygous for the 1758_1759delAG *STAT1* mutation. He developed BCG-osis at the age of 2 mo, which was in remission after antibiotic treatment, followed by recurrent HSV-1 encephalitis from the age of 12 mo until his death at the age of 16 mo. P5 is homozygous for the 1928insA *STAT1* mutation. He developed BCG-osis after vaccination. His genetic, immunological, and clinical features have been reported elsewhere [37]. Informed consent was obtained from all patients and their relatives included in the study.

### Molecular representation of STAT1 phosphodimers.

Models (3-D) of STAT1 mutants E320Q, Q463H, and L706S were built with Modeller software [49]. The X-ray crystallographic structures of STAT1 [32] and STAT3b [50] dimers, available as Protein Data Bank (PDB) entries 1BF5 and 1BG1, were used as templates for comparative modeling [51]. These structures were determined at resolutions of 2.9 and 2.25 Å, respectively, with stretches of missing residues corresponding to regions of low electron density. Before constructing the mutant representations, we therefore built a representation of a complete STAT1 dimer (residues 136–710). We visualized and analyzed 3-D structures with MOLMOL [52].

### Cell culture and stimulation, DNA and RNA extraction, PCR, sequencing, and Northern blotting.

EBV-transformed B cells and SV40-transformed fibroblast cells were cultured as previously described [36]. The parental 2C4 fibrosarcoma cell line and its STAT1-deficient U3C-derived cell line [53] were cultured in RPMI 1640 medium supplemented with 10% heat-inactivated bovine fetal serum (GIBCO-BRL, Paisley, Scotland, United Kingdom). STAT2-deficient U6A cells (derived from the 2fTGH parental fibrosarcoma cell line) [54] were cultured in DMEM supplemented with 10% fetal calf serum. Stimulations were performed with the indicated doses of IFNG (Imukin, Boehringer Ingelheim, Germany) and IFNA2b (IntronA; Schering Plough, Innishannon, Ireland). Genomic DNA and total RNA were extracted from cell lines, fresh blood cells, or tissue biopsy specimens as previously described [36]. Genomic DNA and cDNA were amplified and sequenced as previously described [36]. Primers and PCR conditions are available upon request. Northern blotting was performed as previously described [36]. Sarcoma fibroblast cells were stimulated with 10^5^ IU/ml IFNA or IFNG. The nylon membrane was probed with radiolabeled *IRF1, ISG15, STAT1,* and *GADP* probes (sequences available upon request).

### Determination of mRNA levels by relative Q-RT-PCR.

Total RNA was extracted with Trizol reagent (Invitrogen, Paisley, Scotland, United Kingdom) from EBV-transformed B cells or sarcoma fibroblasts left unstimulated or stimulated with 10^5^ IU/ml IFNA or IFNG. RNA was treated with RNase-free DNase (Roche Diagnostics France, Meylan, France) and cleaned by passage through an RNeasy column (Qiagen S.A. France, Courtaboeuf, France). RNA was then reverse transcribed directly with Oligo-dT, using the TaqMan Reverse Transcription kit (Applied Biosystems, Courtaboeuf, France; and Roche Diagnostics France), for the determination of *MX1, ISG15,* and *IRF1* mRNA levels using the Taqman probes delivered by Applied Biosystems France for these genes. The results were normalized with values for the endogenous *GUS* cDNA.

### Expression vectors and stable transfection.

The mutated *STAT1* alleles were inserted into an M2-tagged pcDNA3-STAT1 vector (a gift from M. J. Holtzman [17]) using the Quickchange site-directed mutagenesis kit (Stratagene, La Jolla, California, United States), following the manufacturer's instructions. U3C cells were cotransfected with one of the various *STAT1* M2-tagged pcDNA3 vectors or an insertless M2-tagged pcDNA3 vector (mock) and the pcDNA3 zeocin-resistant vector, using Lipofectamine 2000 reagent (Invitrogen). The cells were diluted (1:10) 24 h after transfection, and selected 48 h after transfection by culture on medium containing 200 μg/mL zeocin for 10 days, followed by five days of culture on medium containing 1,000 μg/mL zeocin. Living cells were picked and screened by immunofluorescence staining with a STAT1-specific antibody. The pmock clone was selected by pcDNA3 PCR amplification. The populations selected were then cloned by the limiting dilution method. After 1 mo of culture, cells were transferred to medium without zeocin.

### EMSA.

EMSA was carried out as previously described [36]. Briefly, cells were stimulated by incubation for 30 min with 10^5^ IU/ml IFNG or IFNA. We incubated 5–40 μg of nuclear extract with ^32^P-labeled (αdATP) GAS (from the *FCGR1* promoter) or ISRE (from the *ISG15* promoter) probes and subjected the mixture to electrophoresis in a polyacrylamide gel. The additional GAS probes used were taken from the *M67* promoter 5′-*GAT*CCATTTCCCGTAAATCATG*ATC*-3′ and from the *IRF1* promoter 5′-*CTG*ATTTCCCCGAAA*TGA*-3′.

### Immunoprecipitation and Western blotting.

Cells were left unstimulated or were stimulated by incubation with 10^5^ IU/ml IFNG or IFNA for 30 min. For STAT1 immunoprecipitation, whole-cell protein extracts were prepared as previously described [36]. The cell lysates were subjected to immunoprecipitation using SigmaPrep spin columns (Sigma MC1000; Sigma, Saint Louis, Missouri, United States) and 2 μg of anti-STAT1 antibody (TEBU C-111) with 30 μL of protein G (P-3296; Sigma). We washed the immunoprecipitates according to the manufacturer's instructions, and processed them for Western blotting. Western blotting was performed as previously described [36]. The following antibodies were used: anti-phospho-STAT1 (9171; Cell Signaling Technology), anti-STAT1 (610116; BD Transduction Laboratories, San Diego, California, United States), anti-STAT3 (sc-7179; Santa-Cruz Biotechnology, Santa Cruz, California, United States), anti-phospho-STAT2 (4441; Cell Signaling), and anti-STAT2 (sc-476; Santa-Cruz Biotechnology, Santa Cruz, California, United States). Biotinylated peptides were immunoprecipitated as previously described [30]. They were synthesized by Sigma Genosys and immunoprecipitated with streptavidin-agarose (15942–050; Invitrogen). The STAT1 antibody was used for Western blotting, as previously described [36].

### Immunofluorescence.

Immunoflorescence staining was carried out as previously described [17]. The primary antibody was a mouse anti-STAT1 antibody (610116; BD Biosciences, San Diego, California, United States) (1/100 dilution for SV40-transformed fibroblasts and 1/700 dilution for fibrosarcoma clones), and the secondary antibody was an Alexa-488–conjugated goat antimouse IgG (Molecular Probes, Eugene, Oregon, United States) (1/300 dilution for SV40-transformed fibroblasts and 1/1000 dilution for fibrosarcoma clones). The isotypic control was a mouse isotypic antibody (555746; BD Biosciences). Immunofluorescence was detected with a Zeiss Axioplan 2 microscope (Zeiss, Oberkochen, Germany).

### Whole-blood assay of the IL12–IFNG circuit.

Whole-blood assays were performed as previously described [41]. Heparin-treated blood samples from P1, P2, and P3 and their respective controls were stimulated in vitro with BCG alone or with BCG plus IFNG or IL12. Supernatants were collected after 18 and 72 h of stimulation and ELISA was performed with specific antibodies directed against IFNG, or IL12p70, using the human Quantikine IL12p70 HS and the human Pelipair IFNG kit from Sanquin (Amsterdam, the Netherlands), according to the manufacturer's guidelines.

### Viral assays.

Viral assays were performed as previously described [36]. Briefly, skin-derived SV40-transformed fibroblasts, 2C4 and U3C fibrosarcoma cells, and U3C fibrosarcoma–derived clones were left untreated or were treated with 10^5^ IU/mL IFNG for 24 h. They were then infected by incubation with various titers of HSV or VSV for 48 h. Viral titers were then determined by visualizing the lysed wall.

## Supporting Information

Figure S1 GAS-Binding Protein upon IFNA Stimulation EMSAs with the radiolabeled GAS probe of nuclear extracts (5 μg) from patient's EBV-B cells (A) or from a parental fibrosarcoma cell line (2C4) and STAT1-deficient U3C fibrosarcoma cell clones, untransfected (U3C) or stably cotransfected with a zeocin-resistance vector and a vector containing a mock, WT, E320Q, Q463H or L706S *STAT1* alleles (B and C). The cells were not stimulated (NS) or were stimulated for 30 min with 10^5^ IU/ml IFNA (A–C) or IFNG (C). For (A–C), one experiment representative of two to five independent experiments is shown.
(821 KB TIF)

### Accession Numbers

The Online Mendelian Inheritance in Man database (http://www.ncbi.nlm.nih.gov/OMIM) accession number for MSMD is MIM 209950. The RefSeq (http://www.ncbi.nlm.nih.gov/RefSeq) accession numbers for the genes discussed in this paper are STAT1 (6772); IFNG (3458); IFNA (3438); STAT2 (6773); STAT3 (6774); ISGF3G (10379); IFNGR1 (3459); IFNGR2 (3460); JAK1 (3716); JAK2 (3717); TYK2 (7297); IL12B (3593); IL12RB1 (3594); IRF1 (3659); MX1 (4599); ISG15 (9636).

## Abbreviations

BCG: bacille Calmette-Guérin
EBV: Epstein-Barr virus
EMSA: electrophoretic mobility shift assay
GAF: gamma-activating factor
GAS: gamma-activating sequence
HSV: herpes simplex virus
IFNA: interferon alpha
IFNG: interferon gamma
ISGF3: interferon-stimulated genes factor 3
ISRE: IFNA sequence response element
JAK: Janus kinase
MSMD: Mendelian susceptibility to mycobacterial disease
STAT1: signal transducer and activator of transcription-1
SV40: simian virus 40
VSV: vesicular stomatitis virus
WT: wild-type
